# Heterogeneous circRNA expression profiles and regulatory functions among HEK293T single cells

**DOI:** 10.1038/s41598-017-14807-w

**Published:** 2017-10-31

**Authors:** Chaofang Zhong, Shaojun Yu, Maozhen Han, Jiahuan Chen, Kang Ning

**Affiliations:** 1 Key Laboratory of Molecular Biophysics of the Ministry of Education, Hubei Key Laboratory of Bioinformatics and Molecular-imaging, Department of Bioinformatics and Systems Biology, College of Life Science and Technology, Huazhong University of Science and Technology, Wuhan, Hubei 430074 China; 2 Shanghai Center for Systems Biomedicine, Shanghai Jiao Tong University, Shanghai, 200240 China

## Abstract

The single-cell analysis is becoming a powerful method for early detection of the abnormal variant in tissues, especially for profiling a small number of heterogeneous cells. With the advancement of sequencing technologies, many types of non-coding elements including miRNAs and lncRNAs which shed light on their heterogeneous patterns and functions among cells, have been profiled at the single-cell level. However, the complete picture of circRNA profile at single-cell level is still lacking. In this study, RNA-Seq data obtained from single HEK293T cells have been used to analyze expressions and functions of heterogeneous circRNA profiles. The enrichment patterns of circRNAs, interactions with miRNAs and pathways such as ErbB signaling pathway and protein processing in endoplasmic reticulum, have also been investigated. The results showed that circRNAs had a specific distribution pattern which was implicated with expression, miRNA and functional profiles at single-cell level. This assessment study of the expressions and functions of circRNAs at single-cell level shed light on heterogeneities among single cells.

## Introduction

In recent years, with the advancement of next generation sequencing technologies, great progress has been made in transcriptome researches^1–3^, most of which were paid attention to bulk samples. However, intrinsic heterogeneity has been identified to be widespread within the transcriptomes of different individual cells, even within the same types of cells^4,5^. Moreover, previous investigations showed that these genetic heterogeneities might be averaged out in bulk sequencing^6,7^, especially for those rare non-coding RNAs who are dynamically expressed in cells^8^. The single-cell technology can profile heterogeneities within the same tissue at the single-cell level and serve as a powerful method to identify specific properties of each cell^9^. Due to the superiority in detecting single-cell heterogeneity, single-cell technology has become the focus in many fields^9–11^. And a variety of single-cell sequencing methods, such as CEL-seq^12^, Quartz-Seq^13^, Smart-seq^14^, MATQ-seq^15^, make the detection of transcriptional variation in single cells accessible and meet the demand for anatomical resolution. In particular, the study of non-coding RNA at single-cell level has attracted extensive attention^16,17^.

Circular RNA (circRNA) is one of the new members of the non-coding RNA family, forms in a covalently closed continuous loop and isn’t terminated at 5′ and 3′ ends^18^. With the development of high-throughput RNA sequencing technology, abundant circRNAs have recently been identified in many kinds of species and implicated in important functions in physiological and disease process^19–21^. Hence, several comprehensive databases such as CircBase, Circ2Traits^22^, circRNABase^23^, deepBase^24^, and circRNADb^25^, have been developed to merge and unify information of published circRNAs and provide a series of online alignment tools to maintain structure and function prediction. To meet the demand of comprehensive detection of circRNAs, several different pipelines with better performance in circRNA analysis have been developed, including find_circ^19^, CIRI^26^, circRNAFinder^27^, CIRCexplorer^28^, UROBORUS^29^, etc., for further study of circRNAs. However, most of existing work on circRNA is based on bulk sequencing, in which individual cell properties and heterogeneities are hidden. Furthermore, although so much progress has been made in circRNA analysis, neither the circRNA expression pattern nor the specific circRNA function at the single-cell level has been reported. CircRNA analysis at single cell level can yield more detailed and accurate genetic information, and provide a clue for dynamic variation of circRNAs and a new approach for illustrating mechanism and function. To fully reveal the complexity of circRNAs, the circRNA analysis is desirable to be performed at single-cell level.

In this study, to get a high-resolution profile of circRNAs for describing the distribution at the single-cell level, single-cell sequencing datasets were collected. Firstly, we performed expression profile and heterogeneity pattern analysis at single-cell level. Secondly, we elucidated the correlation between the expression of circRNAs and their host genes. Thirdly, by implementing GO analysis, we were interested in the functional enrichment and pathways of the circRNA host genes. Furthermore, we examined the potential function of miRNAs as sponges by inference of circRNA regulatory networks. Our data provided a novel basis for circRNA research in single cells. It was found that the single-cell circRNA profiles might have a specific distribution pattern which was implicated with expression, miRNA and functional profiles at the single-cell level.

## Results

### Detection results of general circRNA by using different methods

We obtained four sets of detection results generated by CIRI2, circRNAFinder, find_circ and CIRCexplorer, respectively. The number of circRNAs ranged from 1,111 to 6,493 in 38 single cells, in which only 410 circRNAs were predicted by all the four methods. In addition, we also detected 68 circRNAs in 7 another single cells from GSE53386. To avoid the technical bias, we focused on the 410 circRNAs found by all the four methods, and also the 68 circRNAs in 7 another single cells were taken into account.

Among circRNAs, heterogeneity was found in each cell. The number of circRNAs in these single cells was significantly different (*t*-test, *p* = 7.338e-15), and the types of circRNAs were also quite different. To characterize the level of heterogeneity, Manhattan distance was used to calculate the distances from the pairwise single cells (Supplementary Fig. 1A). Moreover, heterogeneity was indicated by the hierarchical comparison in each cell and hierarchical clustering showed that heterogeneities of circRNA expression were distinct among cells. In particular, single-cell samples such as SRR5091997 and SRR5091976, which had the similar quantity of sequencing reads, were quite different in circRNAs. Due to the differences of samples, the correlation of samples was analyzed and the hierarchical clustering method was applied to group circRNAs. Samples had only weak correlation or the correlation was not statistically significant (Pearson correlation coefficient (PCC) < 0.32) (Supplementary Fig. 1B), which indicated that circRNAs were fairly independent in single cells.

**Figure 1:**
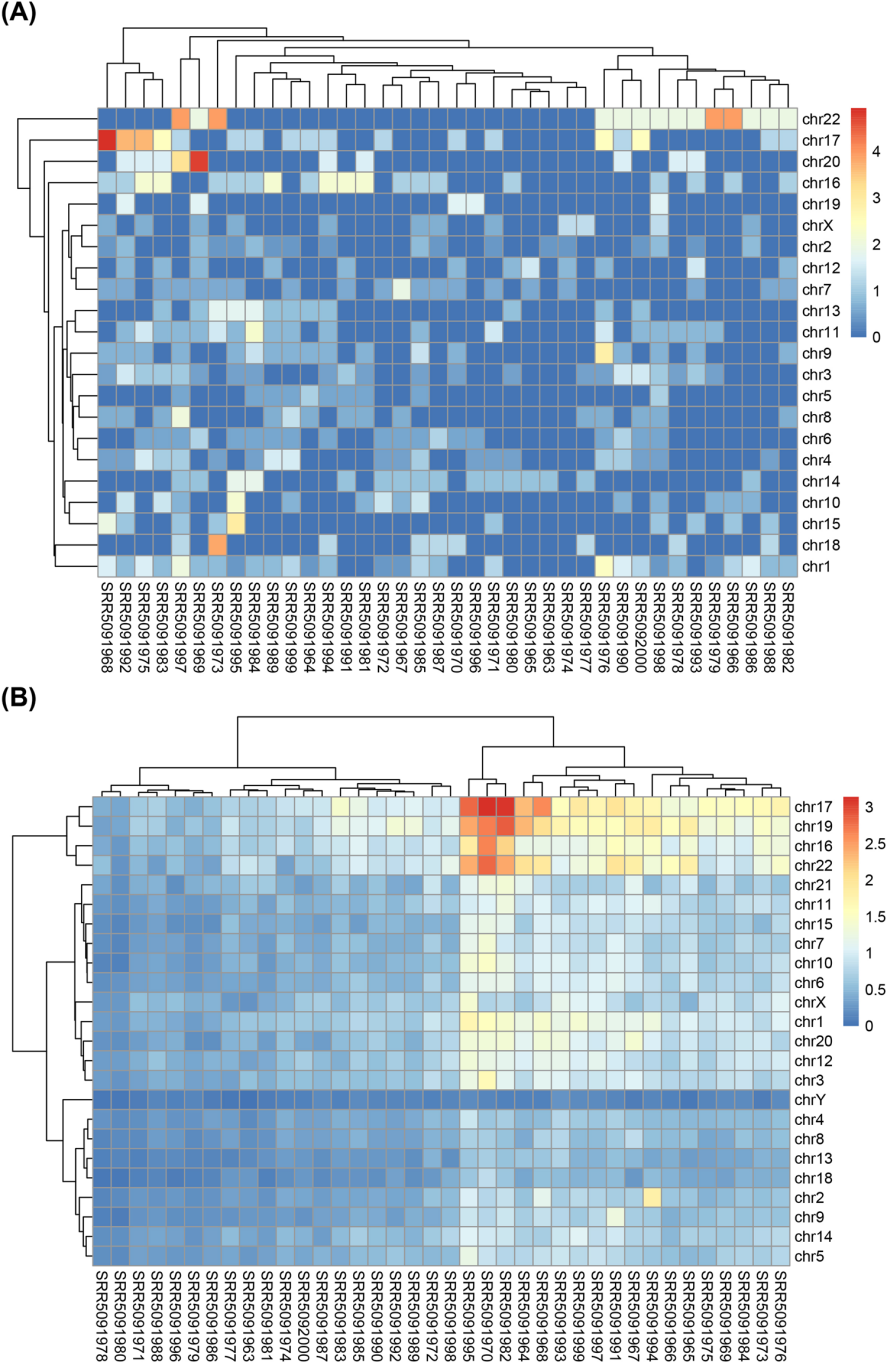
Heatmap of circRNA distribution on the chromosomes in 38 single cells. (**A**) The circRNA enrichment on chromosomes for the 38 single-cell samples; (**B**) Heatmap of the SNP-Freq on each chromosome for 38 single-cell samples obtained by the GATK and Samtools.

The overview of the circRNA distribution and its possible enrichment can be obtained from the circRNA frequencies on each chromosomes. By calculating the ratio of the circRNA counts to the length of chromosome (circRNA-Freq, refer to **Formula (1)** in the **Materials and Methods**), the distribution of the circRNAs on the chromosomes was depicted based on the results from the 38 single cells (Fig. 1A). The Heatmap showed that the distribution of circRNAs on chromosomes was not uniform and enriched on chr22 (*p* < 2.86e-24). In addition, cellular heterogeneity was found among samples, although the cells considered here were cultured in the same condition. The same result was found in 7 single cells from GSE53386 (Supplementary Fig. 2A).

### Comparisons of circRNA and SNP profiles of single cells

**Figure 2:**
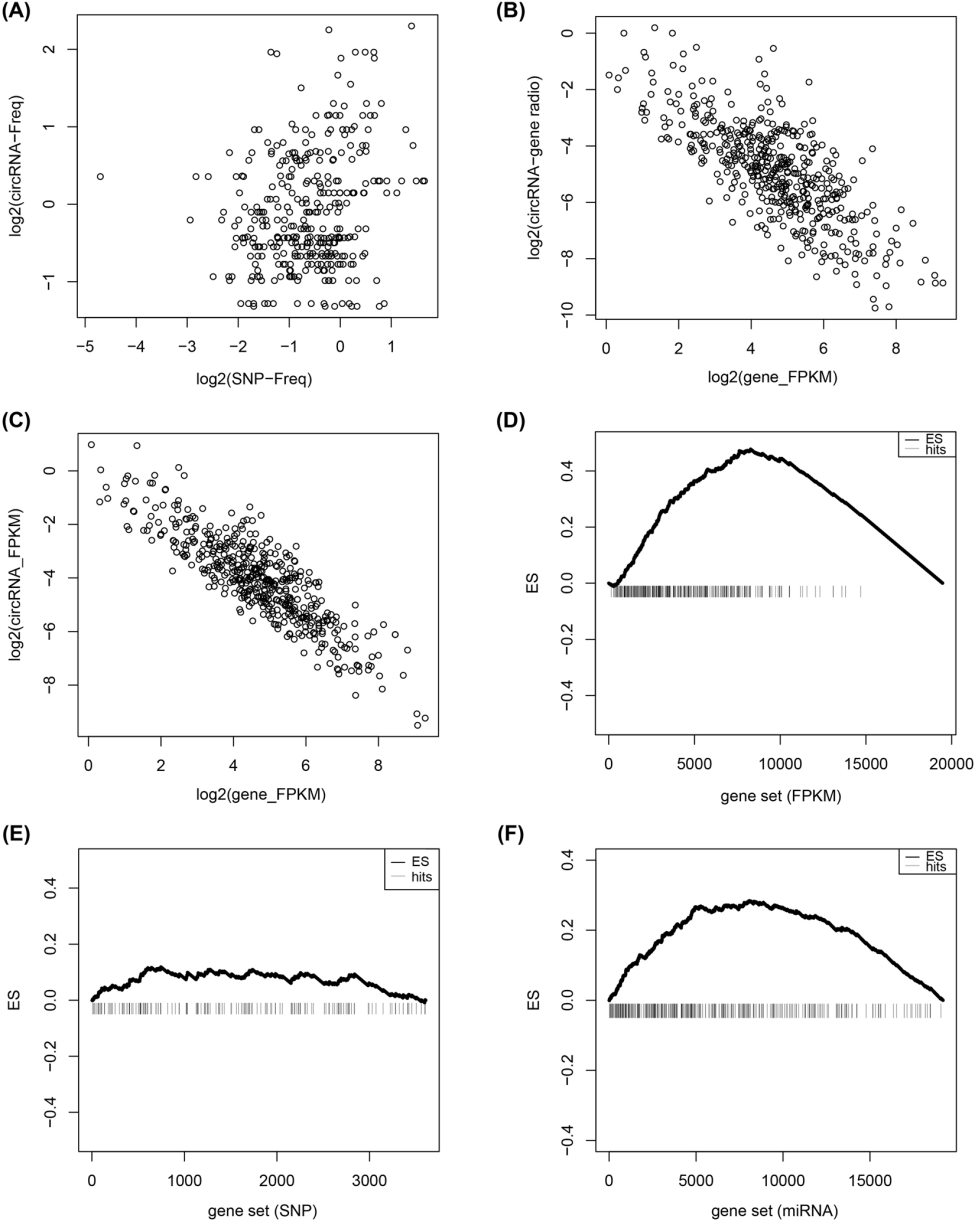
Different degrees of enrichment of expression between circRNAs and their host genes in 38 single cells. (**A**) Correlation analysis for circRNA-Freq and SNP-Freq in 38 single-cell samples; (**B**) Correlation of circRNA-gene ratio and host gene FPKM; **(C)** A negative correlation between expression of circRNAs and host genes; (**D**) The expressed enrichment of circRNA host genes. (**E**) The random distribution of circRNA host genes within the sorted list who contained all the genes with SNPs. (**F**) The enrichment of circRNA host genes sorted by counts of miRNA. The horizontal axes in (**E**), (**D**) and (**F**) represent gene list with expressed gene, SNPs and miRNAs, respectively. And they were ordered by expression level, counts of SNPs and counts of miRNA sites from high to low, respectively. Vertical bars represent the location of circRNA host genes within the sorted lists. The ES values were the maximum deviation from zero encountered in the random walk. The upper curves represent the dynamic ES value.

To find an overview of the SNP distribution and the possible enrichment of SNPs on chromosomes, the SNP frequency on each chromosome was normalized by the quantity of the SNPs and the length of chromosomes (SNP-Freq, refer to **Formula (2)** in the **Materials and Methods**). Each SNP-Freq was calculated and clustered into a heatmap (Fig. 1B). SNPs were distributed widely on 24 chromosomes and enriched on chr16, chr17, chr19 and chr22 (*p* < 4.09e-18). The accumulation of SNPs on chromosomes, accompanied by some genes generating circRNAs such as *CRKL* and *PIK3R3*, were detected to harbor SNPs at different degrees. Obviously, heterogeneity of SNPs was also found among samples. Furthermore, we also analyzed the possible correlation between SNP-Freq and circRNA-Freq, and the result suggested that there was no linear correlation between them (Fig. 2A). The similar SNP profiles and correlation were also found from the GSE53386 (Supplementary Fig. 2B).

Further investigations of correlation of circRNAs and their host genes were discussed based on the expression levels. First, by analyzing the relationship between the circRNA-gene ratio (CGR, refer to **Formula (3)** in the Materials and Methods) and their host gene expression, a strongly negative correlation was presented (PCC = −0.754, *p* < 2.2e-16) (Fig. 2B). Second, the expression levels of circRNAs and their host genes were normalized by FPKM (refer to **Formula (4)** in the **Materials and Methods**). A similar result was observed when we tried to analyze the relationship between gene expression and circRNA expression (PCC = −0.870, *p* < 2.2e-16) (Fig. 2C). Such expamles might indicate that those genes who produced circRNAs with high expression gave rise to the relatively lower expression of circRNAs.

Gene Set Enrichment Analysis (GSEA) was used to demonstrate the enrichment patterns of genes which produced circRNAs. The distribution of circRNA host gene set in three ranked list genes (expressed genes ranked by FPKM, genes with SNPs ranked by quantity of SNP, genes with miRNAs ranked by counts of miRNA) was performed to explore whether these three sets reflect a common distribution of circRNA host genes or not. Firstly, gene expression patterns focused on groups of genes which produced circRNAs from 38 single cells were examined. We observed a significant enrichment of circRNA host genes within highly expressed genes (Fig. 2D), which indicated that the expression patterns of circRNA host genes were consistent and the expression of circRNA host genes was higher than those without circRNA. In contrast, GSEA of circRNA host genes in the gene list with SNP was randomly distributed with poor scores (Fig. 2E), which reflected the relative random of mutation in circRNA host genes. Moreover, circRNA host genes in the gene list with miRNA were enriched at the top of the gene list (Fig. 2F), which mean that genes with circRNAs collectively harbored more miRNA binding sites. A similar conclusion was obtained from the GSE53386 (Supplementary Figs 3, 4).

### Functions of circRNA host genes in single cells

**Figure 3:**
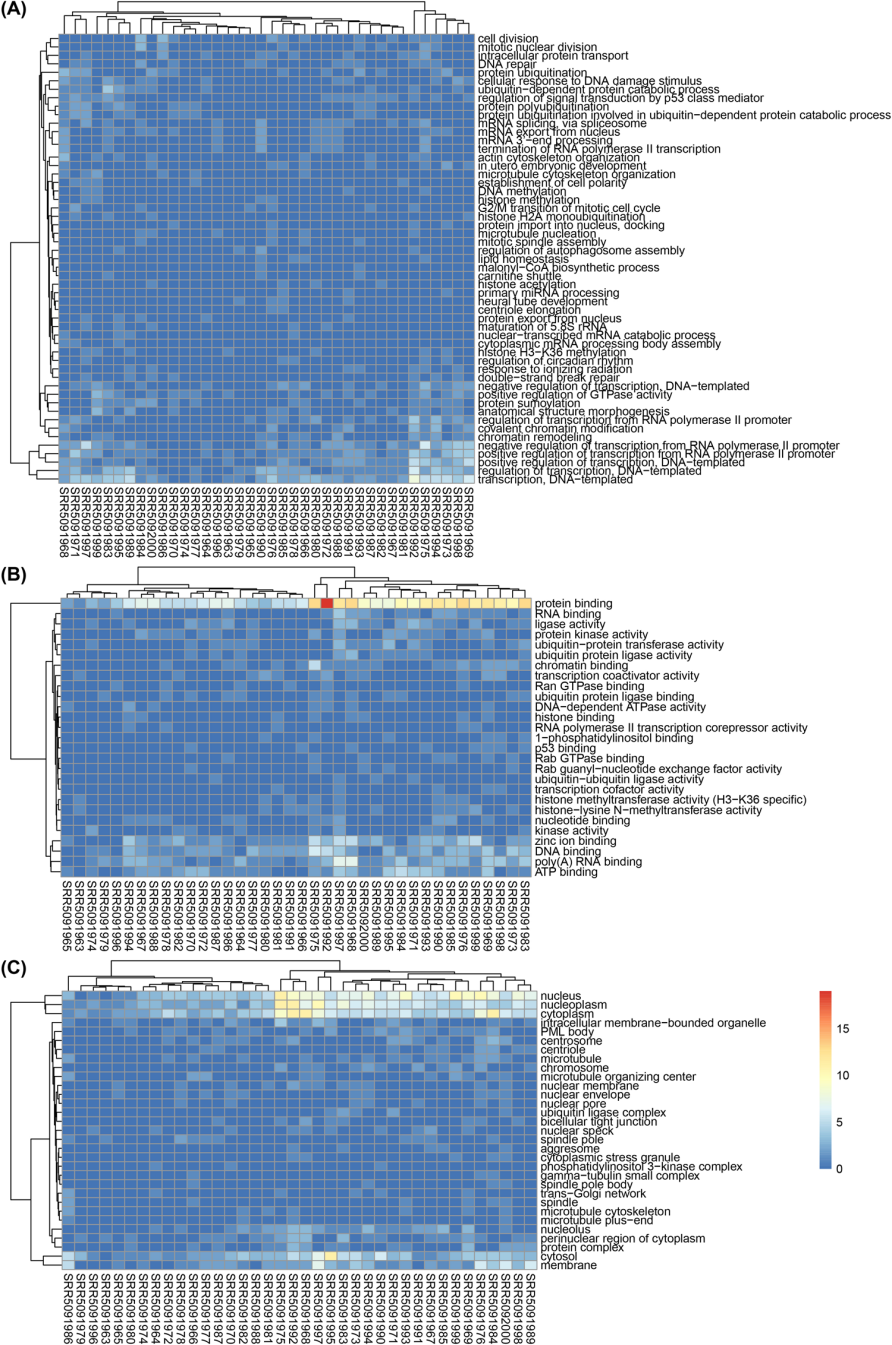
Heatmap of GO terms for the circRNA host genes in 38 single-cell samples. (**A**) CircRNA host gene counts for the GO terms in the Biological Process category; (**B**) CircRNA host gene counts for the GO terms in the Molecular Function category; (**C**) CircRNA host gene counts for the GO terms in the Cellular Component category. Each colored cell in the heatmap represent a standardized number of genes for the GO terms. The cells with high counts are marked in red, and those with low counts are marked in blue.

Gene Ontology annotation can evaluate the function enrichment, as well as to gain an insight into functions of all genes harboring circRNAs. Each gene was associated with at least one GO term and had a wide range of biological functions. According to three categories: biological processes (BP), molecular function (MF) and cellular component (CC), all of GO terms were classified to point out the significantly overlapped functions. In the BP, the circRNA host genes were significantly enriched transcription, DNA−templated, regulation of transcription DNA−templated (*p* < 1.04e-20) (Fig. 3A). In the MF, genes were significantly enriched in protein binding (*p* < 5.56e-31) (Fig. 3B). In the CC, genes were significantly enriched in nucleus, nucleoplasm and cytoplasm (*p* < 1.97e-28) (Fig. 3C). These GO terms associated with circRNA host genes were significantly correlated with the transcription, protein binding, ect., which indicated that those genes were more likely to undergo circularization, and circRNAs might be crucial in the regulation of these functions during embryonic development.

### Pathway analysis of circRNA host genes

**Figure 4:**
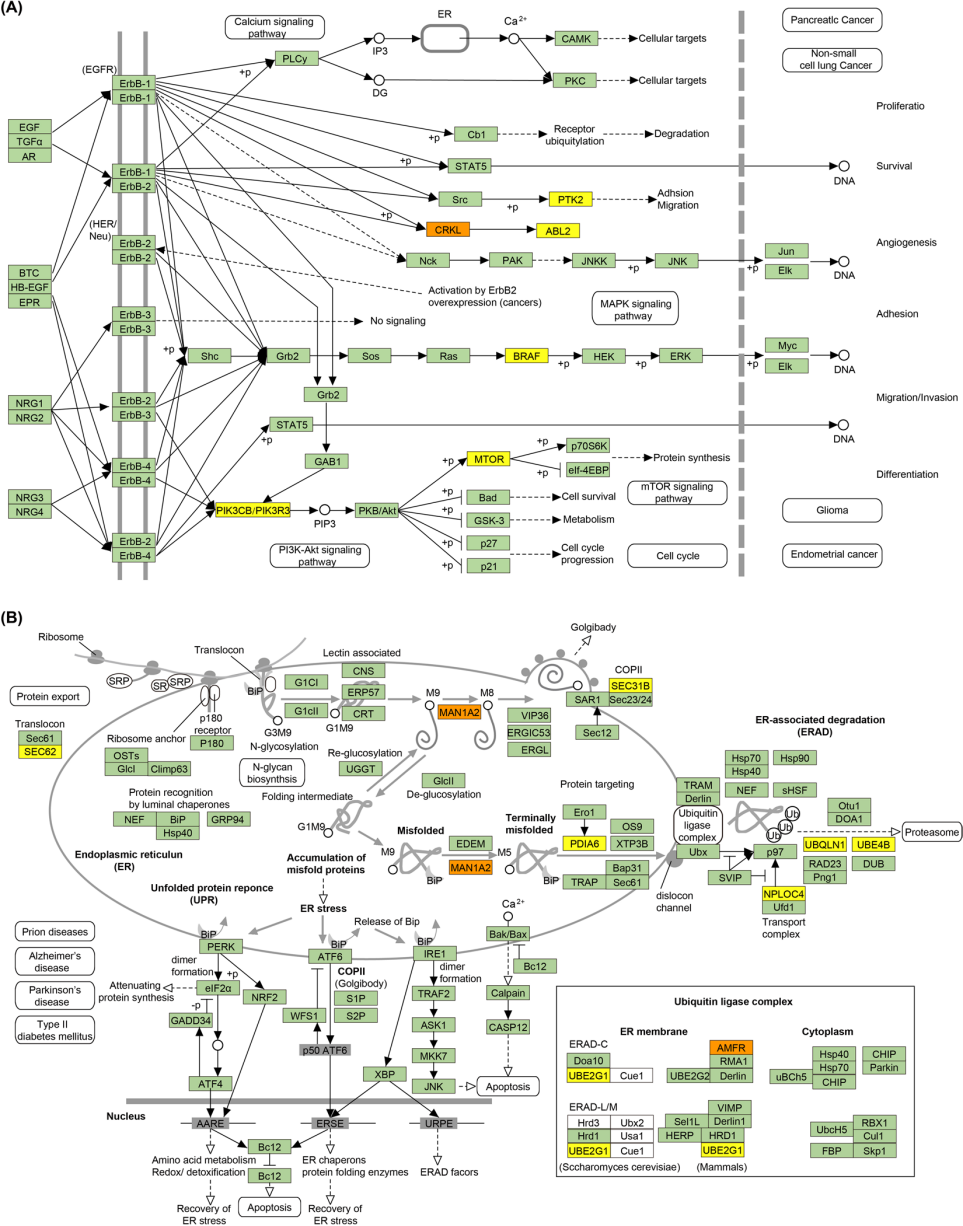
ErbB signaling pathway and its circRNA-related genes. (**A**) The ErbB signaling pathway and its gene interactions; (**B**) Protein processing in endoplasmic reticulum and its gene interactions. The light green boxes represent circRNA-free genes among all single cells, the yellow boxes represent genes harboring circRNAs appearing in only a single cell and the orange boxes represent genes harboring circRNAs appearing in multi-samples.

Genes harboring circRNAs were observed to be involved in different pathways (Table 1), even some of which had a high “sample ratio” (a ratio of samples with at least one circRNA to the total sample). The circRNA host genes *PIK3CB*, *PTK2*, *CRKL*, *PIK3R3*, *BRAF*, *ABL2* and *MTOR* (Fig. 4A) were assigned to the ErbB signaling pathway, and many of them were linked to the enriched GO terms such as protein binding and nucleolus. The ErbB signaling pathway, which regulated diverse physiological responses such as cell survival, proliferation and motility. This consistency between the GO terms and the genes might indicate the possible regulation of these functions in cell metabolism. *CRKL*, which regulated cell adhesion, spreading and migration, harbored circRNAs in 12 cells. And *CRKL* harboring SNP was found in 22 cells. Upon testing the relationship between *CRKL* expression among the cells, we found that the expression of *CRKL* increased (*t*-test, *p* = 0.008) in cells with circRNAs. And also checked the expression of *CRKL* in cells with SNPs and no significant differences existed among the cells. Whether SNPs affected the expression of *CRKL* or not was needed to be further studied.

**Table 1:** Representative circRNA host genes in the pathway analysis.

Pathway	circRNA host genes
Lysine degradation**	*HADH*, *ASH1L*, *SETD2*, *WHSC1*, *NSD1*, *WHSC1L1*, *EHMT1*
Ubiquitin mediated proteolysis**	*HUWE1*, *SMURF1*, *TRIM37*, *UBE4B*, *UBE2G1*, *CUL5*, *BIRC6*, *UBE2I*, *UBR5*, *MID1*
ErbB signaling pathway**	*PIK3CB*, *PTK2*, *CRKL*, *PIK3R3*, *BRAF*, *ABL2*, *MTOR*
Protein processing in endoplasmic reticulum**	*PDIA6*, *SEC31B*, *UBE4B*, *UBQLN1*, *UBE2G1*, *SEC62*, *NPLOC4*, *AMFR*, *MAN1A2*
Insulin signaling pathway**	*PIK3CB*, *CRKL*, *PIK3R3*, *BRAF*, *ACACB*, *ACACA*, *PRKAG2*, *MTOR*
RNA transport*	*NUP214*, *RANGAP1*, *KPNB1*, *CASC3*, *UBE2I*, *EIF4G3*, *NUP205*, *NDC1*
Amoebiasis*	*PIK3CB*, *PTK2*, *PLCB1*, *PIK3R3*, *ADCY1*, *RAB7A*
Selenocompound metabolism*	*MTR*, *PAPSS1*, *MARS*
Bacterial invasion of epithelial cells*	*PIK3CB*, *PTK2*, *CRKL*, *PIK3R3*, *CD2AP*
AMPK signaling pathway*	*PIK3CB*, *PIK3R3*, *ACACA*, *PRKAG2*, *PPARG*, *MTOR*

Note: Genes harboring circRNAs were observed to be involved in ten pathways by p-value cutoff at 0.05. (*p < 0.05, **p < 0.01).

Another pathway protein processing in endoplasmic reticulum, contained circRNA target genes *PDIA6*, *SEC*. *31B*, *UBE4B*, *UBQLN1*, *UBE2G1*, *SEC*. *62*, *NPLOC4*, *AMFR* and *MAN1A2* (Fig. 4B). These genes which played an important role in protein folding, translocation, and degradation, had varying degrees of sample ratios. Among these genes, *MAN1A2* which had Alpha-mannosidases function during the N-glycan maturation process also harbored circRNAs in 12 cells with comparatively higher expression. This circularization in checkpoint genes might have potential effects on their expression regulation and was likely to be important factors in maintaining normal physiological function.

### CircRNA-miRNA-mRNA associations

**Figure 5:**
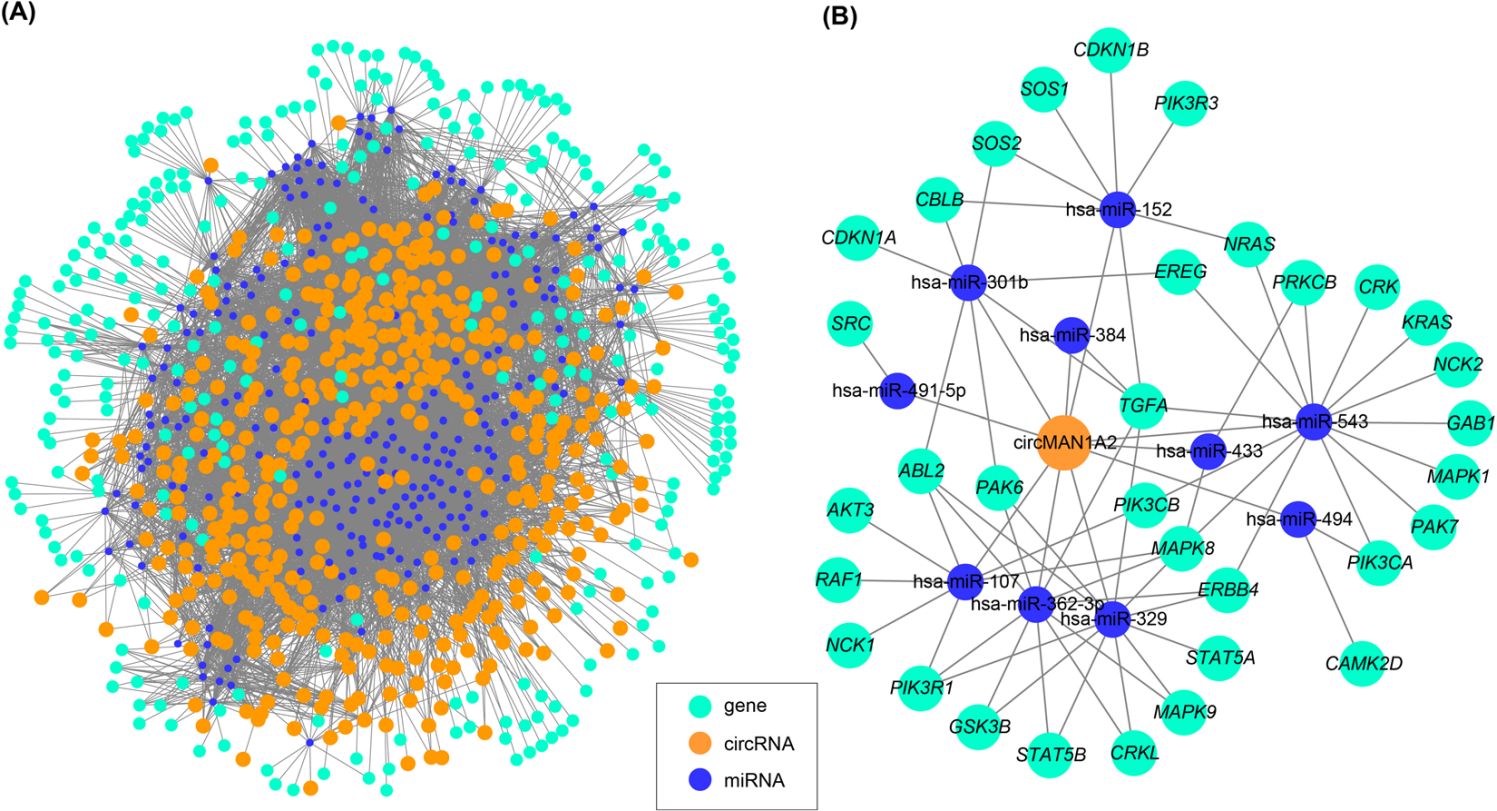
circRNA-miRNA-mRNA networks. (**A**) The circRNA-miRNA-mRNA network, which consists of 406 circRNAs (orange nodes), 249 miRNAs (blue nodes) and 305 disease genes (green); (**B**) The prediction of circMAN1A2 target genes related to ErbB signaling pathway. A total of 44 nodes and 72 edges were constructed.

Interactions, which indicate the functions of circRNAs, between circRNAs and their target miRNAs were predicted according to complementary conserved seed sequence matches. A total of 249 miRNAs could be combined with 406 circRNAs. Among them, the hsa-miR-15a, hsa-miR-15b, hsa-miR-16, hsa-miR-195, hsa-miR-424 and hsa-miR-497 regulated the largest number of circRNAs. Furthermore, investigations on the associations between each potential complementary binding miRNA and their target genes of human diseases were performed. Among the potential complementary binding miRNAs of circRNAs, 120 miRNAs could be associated with a broad spectrum of diseases by acting on 305 disease-related genes. Thus, an entire network of circRNA-miRNA-mRNA interactions was delineated by using Cytoscape (Fig. 5A).

Further, the potential relevance of circRNA and mRNA was established by miRNA to analyze the molecular mechanism. It was predicted that circMAN1A2 could harbor hsa-miR-494, hsa-miR-491-5p, hsa-miR-433, hsa-miR-384, hsa-miR-543, hsa-miR-107, hsa-miR-301b, hsa-miR-329, hsa-miR-152, and hsa-miR-362-3p by miRNAs seed sequence matching, respectively. Moreover, DIANA-miRPath analysis revealed that a total of genes could be regulated by these ten potential miRNAs (Supplementary Table S1), and these target genes were associated with ErbB signaling pathway. As a result, a network of circRNA-miRNA-mRNA interactions in ErbB signaling pathway was established (Fig. 5B).

**Figure 6:**
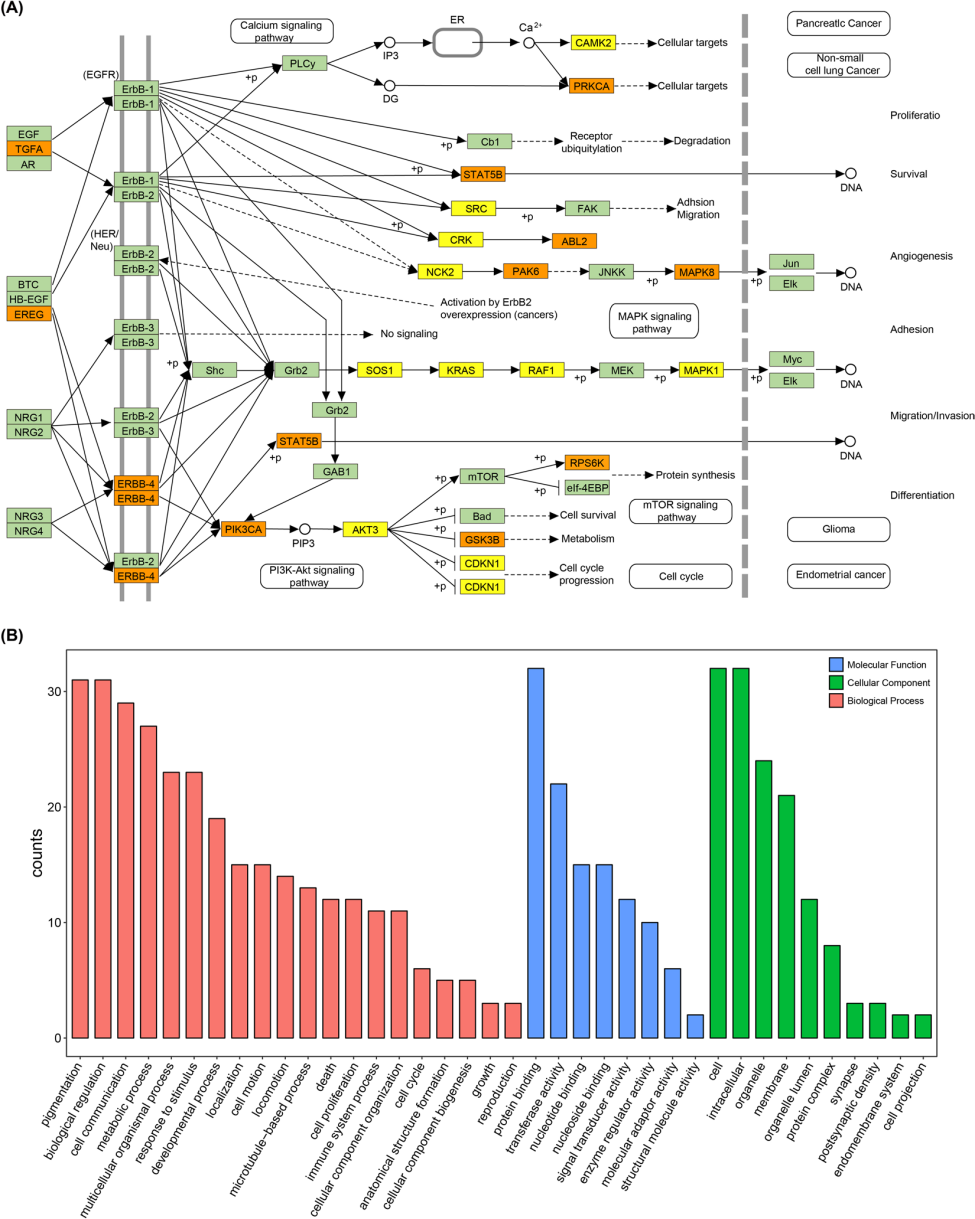
Function annotations for target genes mediated by circMAN1A2-miRNA axes. (**A**) ErbB signaling pathway network containing thirty-three genes on circMAN1A2 mediated by its target miRNAs. The yellow boxes represented target genes containing only one miRNA, the orange boxes represented target genes contained multi-miRNAs; (**B**) GO annotation for the circMAN1A2 targeted genes of the ErbB signaling pathway. Only the most significantly enriched clusters were included.

ErbB signaling pathway network containing 33 genes on circMAN1A2 mediated by hsa-miR-494, hsa-miR-491-5p, hsa-miR-433, hsa-miR-384, hsa-miR-543, hsa-miR-107, hsa-miR-301b, hsa-miR-329, hsa-miR-152, and hsa-miR-362-3p was also established (Fig. 6A). TGFA could be targeted by hsa-miR-384, hsa-miR-543, hsa-miR-301b, hsa-miR-329, hsa-miR-152 and hsa-miR-362-3p in the network, which mean that it might be a crucial factor mediated by circMAN1A2. These findings suggested that circMAN1A2 should participate in the ErbB signaling pathway. When we linked these targeted genes to their GO terms, the GO annotation for target genes revealed that these genes were significantly correlated with pigmentation and biological regulation in BP, cell and intracellular in CC, protein binding in MF (Fig. 6B). This consistency between the GO terms and the ErbB signaling pathway related genes might indicate the possible regulation of these functions in embryonic development at single cells.

## Discussions

In the past few years, circRNA was identified to be highly abundant in mammals based on the study of population cells^30^. However, analysis of population cells could only detect the characteristics of the average, which limited to obtain clues to the differences among each cell^31^. Fortunately, the advent and utilization of single-cell sequencing facilitated the research on heterogeneity and functions of circRNA. In an analysis of publicly available RNA-Seq data from HEK293T single cells, we found that circRNAs were dynamically expressed in single cells.

In this study, we reported the heterogeneity patterns of circRNAs at the single-cell level as well as gene expressions. We obtained 410 circRNAs in 38 single cells, and found that cell-level heterogeneity of circRNAs was obvious, which indicated that circRNA exhibited different expression patterns. These data provided a more comprehensive view into circRNAs than that in published reports in bulk sequencing^32^. The distribution on chromosomes profiles revealed circRNAs were distributed on all chromosomes except for Y chromosome. This distribution was not uniform on the chromosomes and got an enrichment on chr22. And SNPs were widely distributed on 24 chromosomes and were enriched on chr16, chr17, chr19 and chr22. Especially, the possible correlation of circRNA-Freq and SNP-Freq suggested that no correlation between them though SNPs was detected in 166 circRNA host genes. Whether they were relevant or not was still to be further studied. The expression of circRNA had recently been identified to be correlated with that of linear host mRNA, and even for regulate transcription of their host genes^28,33^. In the present study, we had calculated the Pearson correlation coefficient between the expression levels of circRNAs and their host genes. The results showed that a strongly negative correlation between circRNAs and their host mRNA existed, which suggested that those circRNAs have potential influence on the transcription of their host genes. Meanwhile, SNP heterogeneity was also found in 7 single cells from GSE53386.

GSEA was used to reflect enrichment situation of circRNA host genes in three ranked gene lists, including all expressed genes, all genes harboring SNPs and miRNAs. GSEA walked down the ranked list, and the enrichment score was increased if the gene was present in a gene set, otherwise, decreased^34^. The magnitude of the increase or decrease was determined by the correlation of genes with expression level, SNPs and miRNAs. For both GSE78968 and GSE53386 datasets, there were three patterns of circRNAs: (i) the expression of circRNA host genes was higher than those without circRNA; (ii) random distribution in SNPs of the circRNA host genes was observed; (iii) circRNA host genes contained more targeted miRNAs. The common features of the tendency in expression level and miRNA counts had a potential impact on RNA circularization. Therefore, we might conclude that circRNAs might function as miRNA sponges to improve target host gene expression.

Results from GO function annotation and KEGG pathway help to enrich and identify important genes generating circRNAs. GO function annotations for those target genes revealed that they were significantly implicated in protein binding, nucleus, nucleoplasm and cytoplasm, which provided evidence for embryonic development-related circRNA accumulation. Considering KEGG pathway, those circRNA host genes involved important physiological pathways such as ErbB signaling pathway, and protein processing in endoplasmic reticulum, which reflected their importance in embryonic development. Circularization in these checkpoint genes might have potential effects on the ErbB signaling pathway and protein processing in endoplasmic reticulum, which suggested that circRNAs be likely to play regulatory roles in development. As stated in the published study, circRNAs could be correlated with regulation mechanisms of embryonic development^8^.

In addition, we discovered that circRNAs harbored substantially miRNA target sites based on conservative seed sequence matches. Hundreds of circRNA-miRNA interactions were predicted, which would supply new discernment for the underlying mechanisms. CircRNAs could be correlated with disease miRNAs and the circRNA-miRNA axes might participate in disease-related pathways^35,36^. The circRNA-miRNA-mRNA network could serve as the powerful regulation pathway for the cascade amplification effect of circRNA-miRNA and miRNA-mRNA^37^. The best-known biological impact of circRNAs was playing miRNA sponge effects^19^. Therefore, we speculated that circMAN1A2 might competitively bind with hsa-miR-494, hsa-miR-491-5p, hsa-miR-433, hsa-miR-384, hsa-miR-543, hsa-miR-107, hsa-miR-301b, hsa-miR-329, hsa-miR-152, and hsa-miR-362-3p, and had effects on associated target genes. DIANA-miRPath determined the candidate miRNAs of circMAN1A2 which were involved in the ErbB signaling pathway. These miRNA target genes were enriched for functional annotations relating to biological regulation, protein binding, as well as development. Previous studies had revealed that abnormal ErbB signaling in humans was associated with the development of neurodegenerative diseases or a wide variety of types of solid tumor^38–40^. Thus, we predicted that circMAN1A2 could act as a regulator of the ErbB signaling pathway.

CircRNA within single-cells represented one type of the non-coding elements among heterogeneous cells, and we believed that more interesting finding for non-coding element analysis might be revealed based on more complete and accurate single-cell sequencing data. Thus, it was quite promising that the single-cell sequencing was allowing scientists to explore non-coding small molecules diversity in cell populations.

## Conclusion

In this single-cell RNA-Seq analysis of HEK293T cells, not only the circRNA distribution patterns but also the single-cell SNP, gene expression and function profile were profiled. It was found that circRNAs were dynamically expressed in single cells and had obvious heterogeneity by the analysis of distribution patterns. These circRNAs were potentially involved in transcription, DNA−templated, protein binding, and nucleus. Moreover, these circRNAs had a specific distribution pattern which was not associated with SNPs but was implicated with gene expressions or functional profiles at the single-cell level. In addition, the circMAN1A2 might serve as a regulator of the ErbB signaling pathway. These data laid a foundation for further decipher characteristics and regulation mechanisms of circRNAs in single cells.

## Materials and Methods

### RNA-Seq data for the single-cell samples

Two data sets of single HEK293T cells were downloaded from NCBI GEO database (Supplementary Table S2), the first data set (GEO ID: GSE78968) had 38 single cells, which were all obtained from a same HEK293T clone and performed by MATQ-seq. Second data set (GEO ID: GSE53386) had 7 single HEK293T cells, which were performed by SUPeR-seq. Reads of both two data sets were used for the following analyses of circRNA and SNP.

### CircRNA detection

Quality filtering on these reads was performed using Parallel-QC^41^ and sequences which did not fulfill the following criteria were discarded: reads with quality score ≥ 20, the GC proportion ranged from 0.4 to 0.6. All the downstream analyses were based on clean data.

The reference genome and annotation used in the following analyses were downloaded from UCSC Genome Browser (http://genome.ucsc.edu) (hg19 version). Four pipelines, including CIRI, version 2.0.1, circRNAFinder, version 0.1.0, find_circ and CIRCexplorer, version 1.1.10, were introduced to detect the circRNA for the sake of higher accuracy and sensitivity since no specific circRNA detection method has been customized for single-cell RNA-Seq data. All the four pipelines were used the default parameters.

### Manhattan distance calculation

To calculate the degree of heterogeneity in each cell, the Manhattan distance was based on the absence or presence of each circRNA. For each cell, a full (all circRNAs by all cells) 0/1 matrix was built, with “1” denoting presence (defined as the detection of circRNA) and “0” denoting no presence of the corresponding circRNA. Thus, Manhattan distance was used to calculate the distance from the matrix.

### CircRNA enrichment on chromosomes

For circRNA enrichment on chromosomes, the circRNA frequency on each chromosome was normalized by the following formula:

1 $$circRNA-Freq={10}^{8}\times \frac{circRNA\,counts/Chromosome}{chromosome\,length}$$

Where circRNA counts/Chromosome was the quantity of the circRNAs detected on one chromosome, and chromosome length was the length of this chromosome. The factor 10^8^ was chosen as the denominator to leverage the circRNA-Freq values for a fair and easy comparison.

### SNP identification

Reads were mapped to the human genome using BWA software, version 0.7.15^42^. The variants calling was performed in GATK^43^ and Samtools, version 1.3^44^. HaplotypeCaller was used for variant calling in GATK while mpileup and view were used in Samtools. The overlaps of GATK and Samtools results were considered as candidates, which were subjected to additional filtering to remove SNPs with low-quality value (QUAL < 30), low QD (QD < 20.0), low read coverage (DP < 8) and Strand Bias (FS > 30.0).

For SNP enrichment on chromosomes, the SNP frequency on each chromosome was normalized using the following formula:

2 $$SNP-Freq={10}^{6}\times \frac{SNP\,counts/Chromosome}{chromosome\,length}$$

Where SNP counts/Chromosome was the quantity of the SNPs detected on one chromosome, and chromosome length was the length of this chromosome. The factor 10^6^ was chosen as the denominator to leverage the SNP-Freq values for a fair and easy comparison.

### Expression analysis

RNA-seq libraries were mapped to the reference genome using tophat, version 2.1.0^45^, after that the FPKM values for each gene were calculated by cufflinks, version 2.2.0^46^ and reads mapped to genomic features were counted using htseq-count. Therefore, to estimate abundance of circRNAs, the circRNA-gene ratio (CGR) was quantified using the following formula:

3 $$CGR(circRNA)=\frac{junction\,spanning\,reads}{host\,gene\,{\rm{reads}}}$$

To generate an overview of circRNA expression profiles among the single cells, the hierarchical clustering analysis was performed based on expression value of all target circRNAs. Expression levels of circRNAs were quantified by the number of junction spanning reads. To obtain an estimate of relative expression, the number was normalized to the total number of reads in the library and the host gene FPKM. The FPKM of circRNA was calculated using the following formula:

4 $$FPKM\,(circRNA)=\frac{junction\,spanning\,reads}{mapped\,reads\,(millions)\times host\,gene\,FPKM}$$

Where junction spanning reads was the amount of the circRNAs detected on one site, and million mapped reads were the total reads which mapped to reference genomes.

### miRNA analysis

For miRNA analysis, microRNA target prediction in human was available on Miranda database (http://www.microrna.org/microrna/home.do), which was downloaded to evaluate all instances of conservative sites in each gene. The miRNA-disease associations were predicted in the Human MiRNA Disease Database (HMDD http://cmbi.bjmu.edu.cn/hmdd). All mature miRNA sequences were downloaded from miRBase (http://www.mirbase.org), each circRNA was scanned to identify miRNA target sites based on conserved seed sequence matches. The miRNA pathway investigating was carried out based on DIANA-miRPath by *p*-value cutoff at 0.05. The graphs of the circRNA-miRNA and circRNA-miRNA-gene networks were visualized on Cytoscape, version 3.3.0^47^.

### Functional enrichment analysis

The function of those target genes was predicted and annotated in the network by Database for Annotation, Visualization and Integrated Discovery (DAVID https://david.ncifcrf.gov), P < 0.05 was used as the criterion for statistical significance. Gene Ontology (GO) that describes genes from any organism were used. GO Terms were classified into three categories: biological processes (BP), cellular component (CC) and molecular function (MF). Pathway analysis was carried out for a functional analysis of mapping genes to KEGG^48^ pathways.

## Electronic supplementary material

Supplementary file
