# Supplementary material for: Heterogeneous circRNA expression profiles and regulatory functions among HEK293T single cells
Source: Sci Rep. 2017 Oct 31;7:14393. doi: 10.1038/s41598-017-14807-w (PMC5663837; doi:10.1038/s41598-017-14807-w)
Supplement: Supplementary file 1 — Supplementary file [file 41598_2017_14807_MOESM1_ESM.doc]

**Supplementary file for “Heterogeneous circRNA expression profiles and regulatory functions among HEK293T single cells”**

Chaofang Zhong^1^, Shaojun Yu^1^, Maozhen Han^1^, Jiahuan Chen^2^, Kang Ning^1,*^

*^1^Key Laboratory of Molecular Biophysics of the Ministry of Education, Hubei Key Laboratory of Bioinformatics and Molecular-imaging, Department of Bioinformatics and Systems Biology, College of Life Science and Technology, Huazhong University of Science and Technology, Wuhan, Hubei 430074, China*

*^2^ Shanghai Center for Systems Biomedicine, Shanghai Jiao Tong University, Shanghai, 200240, China*

*^*^* Corresponding author*.* E-mail: [ningkang@hust.edu.cn](mailto:ningkang@hust.edu.cn)

**Supplementary Figures**


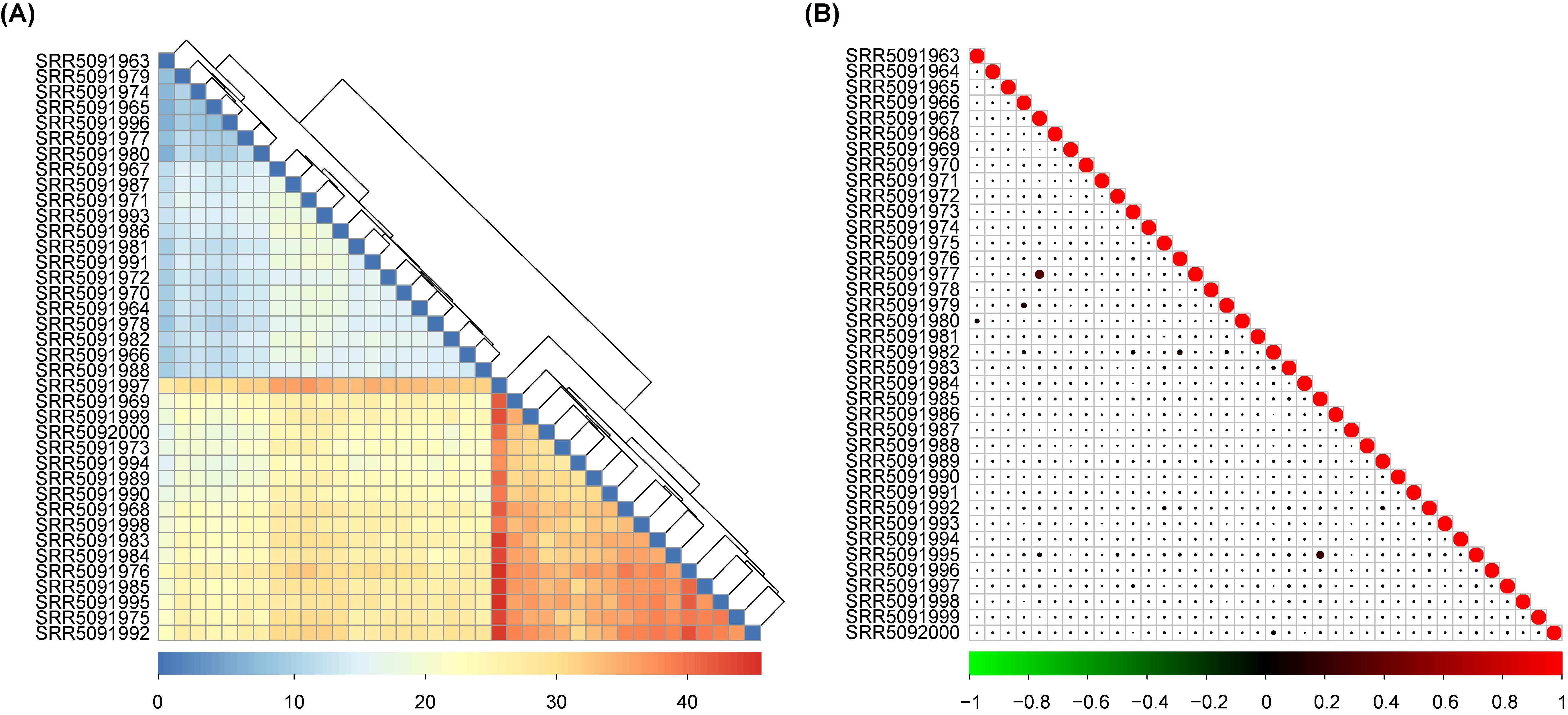


**Supplementary Figure 1. Pairwise comparisons of 38 single cells from GSE78968. (A)** Manhattan distance from the pairwise single cells based on the absence or presence of each circRNA; **(B)** Pairwise comparisons of circRNAs of each cell, with a color gradient denoting Pearson Correlation Coefficients. Samples were only weakly correlated or the correlations were not statistically significant.


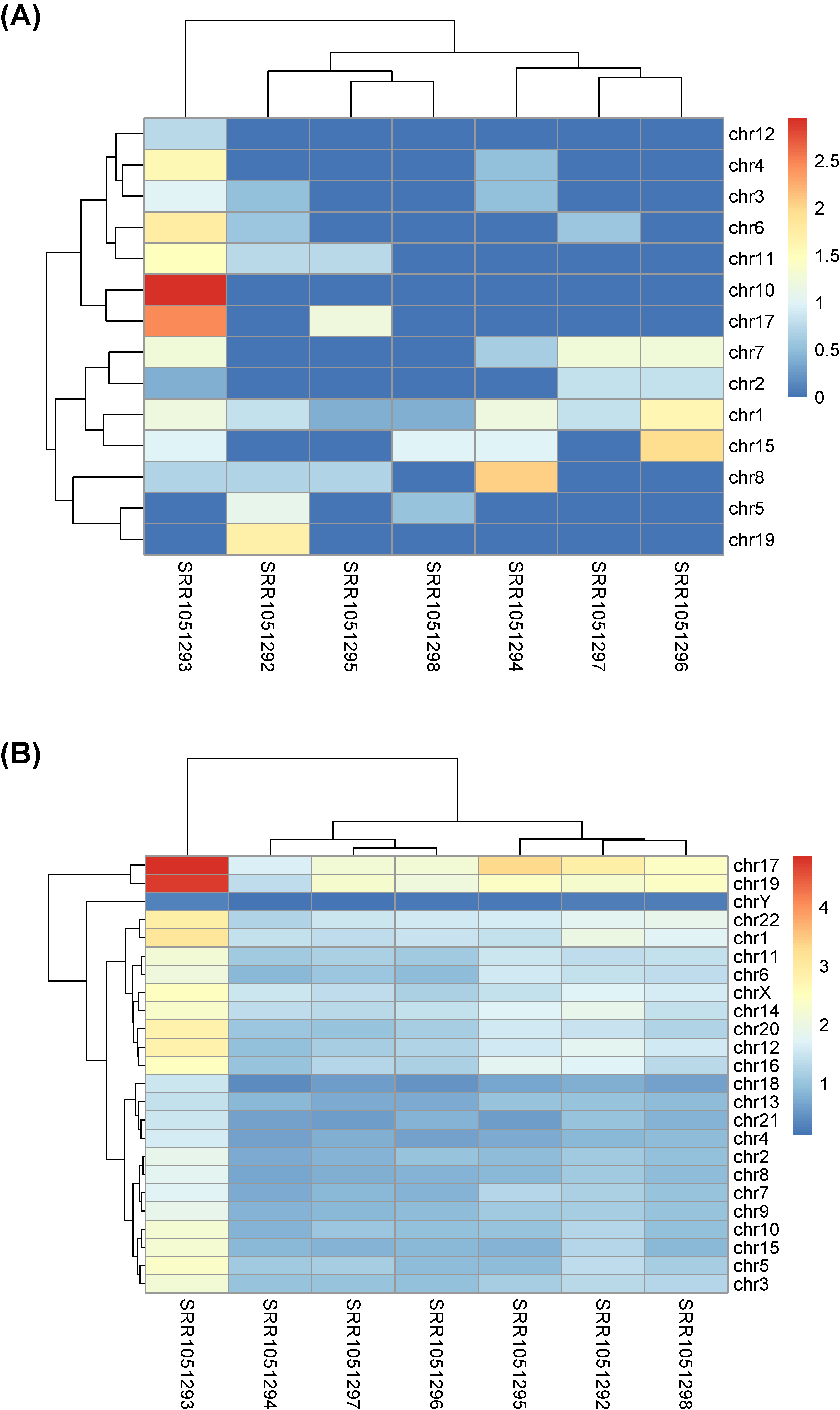


**Supplementary Figure 2. The distribution of the circRNAs on the chromosomes in 7 single cells from GSE53386.** **(A)** The circRNA enrichment on chromosomes for the 7 single-cell samples; **(B)** Heatmap of the SNP-Freq on each chromosome for 7 single-cell samples obtained by the GATK and Samtools.


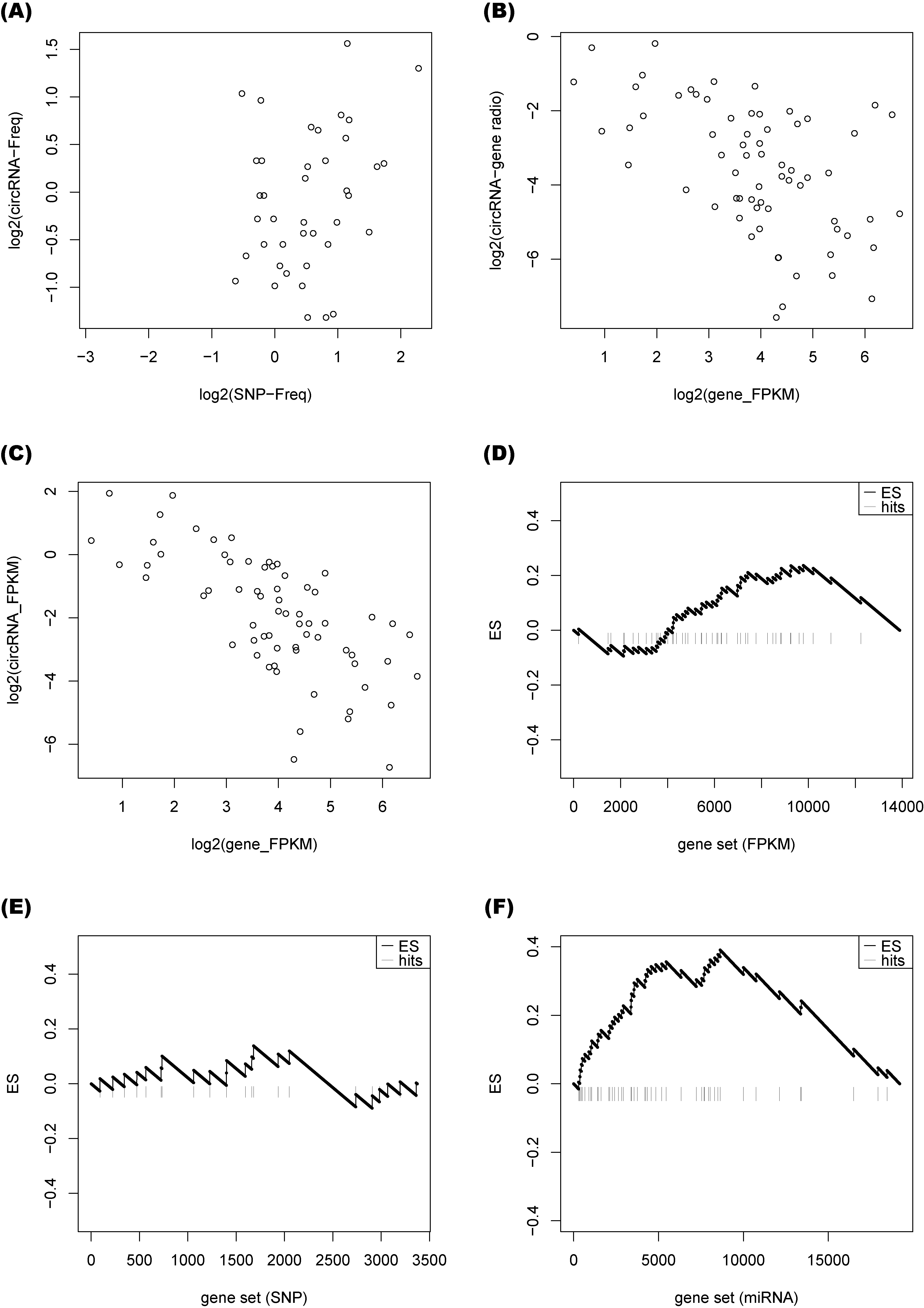


**Supplementary Figure 3. Different degrees of enrichment between circRNAs and their host genes in 7 single cells from GSE53386. (A)** Correlation of circRNA-Freq and SNP-Freq; **(B)** Correlation of CircRNA-gene ratio and host gene FPKM; **(C)** Correlation of expression between circRNAs and host genes; **(D)** The enrichment of circRNA host genes; **(E)** The random distribution of circRNA host genes within the sorted list containing all the genes with SNPs; **(F)** The circRNA host genes enriched on the top of the sorted list. The RNA expression analysis was performed by TopHat and Cufflinks.


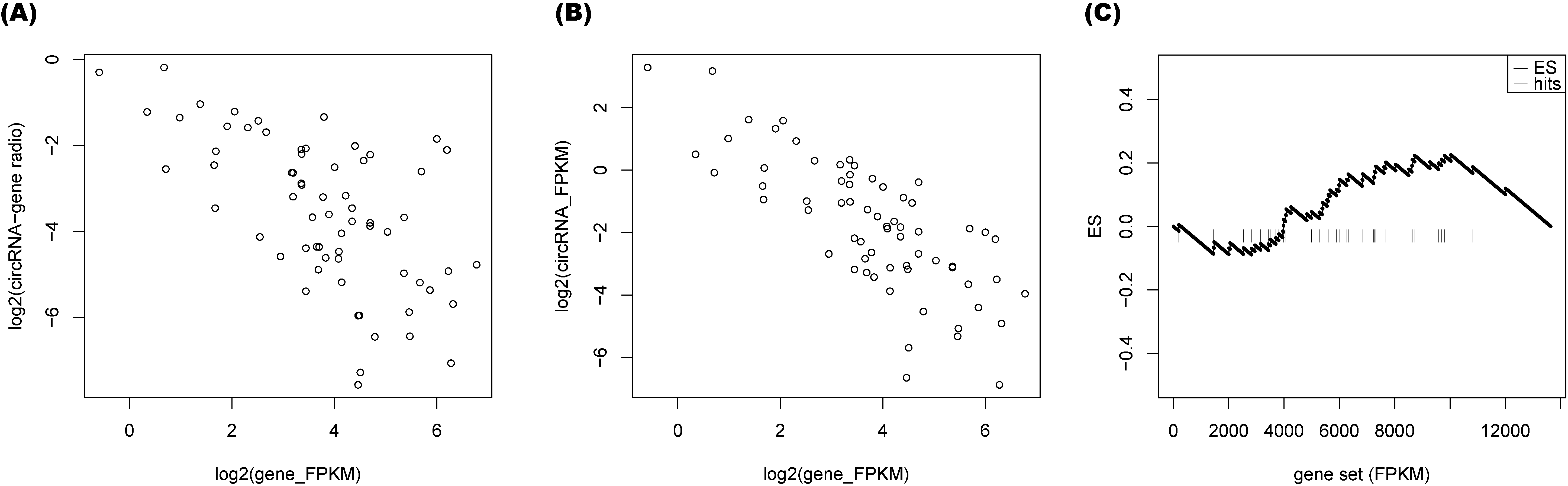


**Supplementary Figure 4. Different degrees of enrichment between circRNAs and their host genes in 7 single cells from GSE53386 using the STAR. (A)** Correlation of circRNA-gene ratio vs host gene FPKM; **(B)** Correlation of expression between circRNAs and host genes; **(C)** The enrichment of circRNA host genes.

**Supplementary Tables**

**Supplementary Table S1. Target genes of the potential miRNAs of circMAN1A2.**

**Supplementary Table S2.** **Information of the single cells from two datasets (GSE78967 and** **GSE53386) used in the study.**
